# An integrated targeted metabolome of phytohormones and transcriptomics analysis provides insight into the new generation of crops: *Polygonatum kingianum* var. *grandifolium* and *Polygonatum kingianum*

**DOI:** 10.3389/fpls.2024.1464731

**Published:** 2024-09-24

**Authors:** Luyun Ning, Qian Xiao, Chensi Tan, Limin Gong, Yeman Liu, Zhi Wang, Shujin He, Chengdong He, Hanwen Yuan, Wei Wang

**Affiliations:** ^1^ Traditional Chinese Medicine (TCM) and Ethnomedicine Innovation and Development International Laboratory, Innovative Material Medical Research Institute, Hunan University of Chinese Medicine, Changsha, China; ^2^ School of Pharmacy, Hunan University of Chinese Medicine, Changsha, China; ^3^ Production Department, Hunan Xinhui Pharmaceutical Co., Ltd, Changsha, China

**Keywords:** *Polygonatum kingianum* var. *grandifolium*, *Polygonatum kingianum*, Huangjing, high-yield, autumn shooting, phytohormone

## Abstract

Huangjing is becoming a new generation of crop. *Polygonatum kingianum* var. *grandifolium* (XHJ) is a variant of *P. kingianum* (DHJ), and they are treated as Huangjing. Unlike other *Polygonatum* species, the rhizome bud of XHJ can germinate both in spring and autumn, which contributes to its high rhizome yield. However, the molecular mechanism of the autumn shooting of XHJ was still unknown. In the present study, cellular observation, comparative targeted metabolome of phytohormones, and transcriptome analysis between XHJ and DHJ in autumn were conducted. Interestingly, ‘Diterpenoid biosynthesis’ (ko00904) and ‘Plant hormone signal transduction’ (ko04075) were commonly enriched by differentially accumulated phytohormones (DAPs) and differentially expressed genes (DEGs) in all tissues, which indicated the high auxin content, low cytokinin (CTK) content, and low abscisic acid/gibberellin (ABA/GA) ratio might contribute to the XHJ rhizome buds’ differentiation and germination in autumn. Moreover, according to the weighted gene co-expression network analysis (WCGNA), transcript factors (TFs) related to auxin, CTK, GA, and jasmonic acid (JA) metabolism were screened, such as *AP2/ERFs*, *WRKY*, and *NAC*, which deserve further research. In conclusion, we comprehensively illustrated the mechanism of XHJ natural autumn shooting through cytological, metabolic, and transcriptomic analysis, which improves our understanding of the high yield of XHJ rhizomes and the diversity of shooting mechanisms in *Polygonatum* to lay the foundation for the further development of the Huangjing industry.

## 1 Introduction

According to the Chinese Pharmacopoeia, Polygonatum cyrtonema, *Polygonatum sibiricum*, and *Polygonatum kingianum* are the original plants of the traditional Chinese drug ‘Huangjing’ (i.e., *Polygonati rhizoma*). All of them are perennial herbs with a rhizome combining edible and medicinal values and have broad prospects for utilization and development (Luo et al., 2022; Zeng et al., 2020). Many compounds have been identified from Huangjing, including polysaccharides, saponins, flavonoids, lignans, amino acids, and trace elements, which contribute to its antioxidant, cytotoxic, anti-inflammatory, and anti-fatigue activities (Deng et al., 2024; Luo et al., 2022; Zeng et al., 2020). The development of Huangjing has mainly been in health products, including preliminarily processed products, for example, steaming and processing Huangjing for Huangjing cookies, Huangjing wines, Huangjing drinks, Huangjing pills, and Huangjing yogurts (Li et al., 2023; Deng et al., 2024). Moreover, more deeply processed Huangjing products have also been explored, such as a sweetened roll of *rhizoma polygonati*, hawthorn, and yam (Sun et al., 2023). Currently, using agricultural biodiversity to develop a new generation of crops that are both nutritious and high-yield has become an international trend, and Huangjing has notable characteristics and advantages (Si et al., 2024).

*P. kingianum* var. *grandifolium* (XHJ) is the variant of *P. kingianum* (DHJ). It has been treated as Huangjing in folk medicine and has been largely cultivated in China (Shi et al., 2022; Yu et al., 2022). In previous studies, the total polysaccharide content of XHJ rhizome from Xiushan County, Chongqin City and Enshi City, Hubei Province was 134.04 mg/g and 84.3 mg/g respectively (Yu et al., 2022; Liang, 2023), which was much higher than 70 mg/g (the quality standard of Huangjing according to the Chinese Pharmacopoeia). Liang (2023) indicated that the total polysaccharide of XHJ had good antioxidant activity and an inhibitory effect on tumor cells; XHJ also contained higher amino acids, which might contribute to the anti-inflammatory and anti-diabetic activity of XHJ (Liang, 2023).

Generally speaking, *Polygonatum* species are perennial and the rhizome buds only germinate in spring, and the overground part dies away in autumn. However, XHJ germinates twice in 1 year (once in spring and once in autumn), so XHJ stays evergreen all year round (Shi et al., 2022; Yu et al., 2022). Thanks to this biological characteristic, the total photosynthesis time of XHJ is much longer than other *Polygonatum* species, so the rhizome yield of XHJ is the highest (Shi et al., 2022). After 5 years of efficient planting, the yield of XHJ can reach more than 5,000 kg per mu (Shi et al., 2022). Hence, revealing the molecular mechanism of the special autumn shooting habit in XHJ would be helpful in uncovering its high-yield mechanism.

For perennial plants, dormancy is an adaptive mechanism formed in the long-term evolution process, which is very important for surviving in the unfavorable growing season and is a beneficial biological habit (Lubzens et al., 2010). When the harsh environment (such as high temperatures in summer or low temperatures in winter) comes, plants can sense the changes in the external environment, and form a storage organ or protective structures with meristem ability (such as seeds, buds, and other abnormal organs), and show a temporary stop in growth (Lang et al., 1987). The newborn rhizome buds of most *Polygonatum* species in the current year stay dormant in winter and germinate in the next spring to overcome the low temperatures in winter. However, after a hot summer, the temperature falling in autumn seems a signal that induces the germination of XHJ rhizome buds, which is significantly different from other *Polygonatum* species.

Phytohormones are closely related to the bud formation, dormancy, and germination processes and include auxin, cytokinin (CTK), gibberellin (GA), abscisic acid (ABA), and ethylene (ETH) (Wei et al., 2022). Different phytohormones are induced by different chemical or physical signals and play different roles in bud dormancy. Moreover, the same hormone plays different functions during bud dormancy production, maintenance, and release (Wojtania et al., 2022).

In the present study, cellular observation, UPLC-MS/MS-based targeted metabolomic analysis of phytohormones, and RNA-seq were conducted, which improve our comprehensive understanding of the diversity of shooting mechanisms between XHJ and DHJ in autumn, and provide insight into the high-yield mechanism of XHJ to enhance the development of the Huangjing industry.

## 2 Materials and methods

### 2.1 Plant material

XHJ was collected from Chetou Village, Lufeng Town, Xupu County, Hunan Province, China. DHJ was collected from Qinglong Town, Jianshui County, Yunnan Province, China. All these plants were transplanted into the medicinal plant garden of Hunan University of Chinese Medicine in January 2022, and the rhizome, roots, and buds were collected in September 2022, when XHJ began to germinate. These tissues were frozen in liquid nitrogen immediately and stored at -80 °C. All of these plants were identified as *Polygonatum kingianum* var. *grandifolium* and *Polygonatum kingianu*, respectively by Associate Professor Yeman Liu (Hunan University of Chinese Medicine). The authenticated specimens of *Polygonatum kingianum* var. *grandifolium* (Code: 20220902-1) and *Polygonatum kingianum* (Code: 20220902-2) were kept in the TCM and Ethnomedicine Innovation & Development International Laboratory, Innovative Materia Medica Research Institute, Hunan University of Chinese Medicine.

### 2.2 Observation of paraffin sections

The buds from XHJ and DHJ were vacuum-infiltrated and fixed with 50% FAA (v/v) and were also collected in September 2022. The procedure for paraffin section observation was followed as described in our previous study (Ning et al., 2019).

### 2.3 Total polysaccharide content

Total polysaccharide was extracted and tested from the dried XHJ and DHJ rhizomes as in the method described in the Chinese Pharmacopoeia. Three repetitions were conducted in each sample and statistical analysis was performed by SPSS 22.0 software.

### 2.4 Phytohormones analysis

The rhizome, roots, and buds of XHJ and DHJ were chosen for targeted metabolomics of phytohormones analysis, which were abbreviated as X-rhizome, X-root, X-bud, D-rhizome, D-root, and D-bud, respectively. Each group had three biological replicates, and each biological replicate contained more than 10 plants. This part of the experiments was carried out by MetWare Biotechnology Co., Ltd. (Wuhan, China). In total, 88 phytohormones were detected, including 2 ABAs, 26 auxins, 36 CTKs, 10 GAs, 9 jasmonic acids (JAs), 2 salicylic acids (SAs), 2 strigolactones (SLs) and 1 ethylene (ETH). All samples were vacuum-freeze dried and ground to powder separately. The sample extracts were analyzed by a UPLC-Q-TOF-MS/MS system (ExionLC™ AD, https://sciex.com.cn/, QTRAP^®^6500+, https://sciex.com.cn/). The conditions for the high-performance liquid chromatography (HPLC) were as follows: chromatographic separation was conducted on Waters’ ACQUITY UPLC HSS T3 C18 column (1.8 µm, 100 mm × 2.1 mm), using water with 0.04% acetic acid and acetonitrile with 0.04% acetic acid as mobile phase A and B, respectively. The elution program followed was: 95:5 V(A)/V(B) at 0 min, 95:5 V(A)/V(B) at 1 min, 5:95 V(A)/V(B) at 8 min, 5:95 V(A)/V(B) at 9 min, 95:5 V(A)/V(B) at 9.1 min, and 95:5 V(A)/V(B) at 12 min. The flow rate was 0.35 mL/min, the column temperature was 40 °C, and the injection volume was 2 μL. Mass data acquisition was carried out in ion spray voltage in positive (5500 V)/negative (-4500 V) mode, with an electrospray ionization temperature of 550 °C. Curtain gas was set at 35 psi. The content of phytohormones was determined using an internal standard method.

Principal component analysis (PCA) of different samples for comparison was conducted by ‘prcomp’ in R software (www.r-project.org/). The DAPs were determined by log2 fold change ≥ 2 or ≤0.5 and variable importance in projection ≥ 1. The identified DAPs were further analyzed using the KEGG database.

### 2.5 Transcriptomic analysis

The X-rhizome, X-root, X-bud, D-rhizome, D-root, and D-bud were also used for transcriptomic analysis. Each group had three biological replicates, and each biological replicate contained more than 10 plants. A TRIzol RNA extracting kit was used to extract the total RNA. RNA concentration and integrity were detected. The mRNAs with polyA tails were enriched by Oligo(dT) beads. The double-stranded cDNA was purified using AMPure XP beads and repaired at the end: adding an A-tail and the sequencing linkers. A cDNA library was constructed, and the Illumina Novaseq6000 system platform was used to sequence. CASAVA base recognition was conducted to convert the image data into raw data. The fastq software was used to obtain clean reads (Chen et al., 2018), and the clean reads were stitched using the Trinity software (Grabherr et al., 2011). The transcriptome’s overall quality was assessed for completeness using the Benchmarking Universal Single-Copy Orthologs tool (BUSCO version 5.4.3) (Seppey et al., 2019). The gene expression levels were measured by FPKM (Chen et al., 2021). DESeq2 software was used to identify the DEGs with p-values < 0.05 and |log2 fold changes| ≥ 1. The KEGG database was used to perform the DEG enrichment analysis.

### 2.6 qRT-PCR

ReverTra Ace^®^ qPCR-RT Master Mix with gDNA Remover (TOYOBO) was used for total RNA reverse transcription according to the manufacturer’s protocol. *Elongation factor 1-α* was used as an internal reference (Li D. D. et al., 2022). Specific primers were designed by Primer 5.0 software. An Applied Biosystems QuantStudio 5 system with the Hieff^®^ qPCR SYBR Green Master Mix (Low Rox Plus) (YEASEN) was used to perform the qRT-PCR. The relative expression was calculated by the 2^-ΔΔCt^ method (Livak and Schmittgen, 2001). All primer sequences are listed in **Supplementary Table S1**.

### 2.7 Weighted gene co-expression network analysis

The DEGs identified by the transcriptomic analysis were used to obtain the co-expression network modules by the WGCNA package in R. The co-expression modules were generated via the automatic network construction function with the default parameters: mergeCutHeight was 0.25, powerEstimate was 18, and minModuleSize was 50. The eigengene values were calculated in each module, which was applied to search for the association with the main compounds in XHJ and DHJ. The module member-ship value was obtained by using the signedKME function of the WGCNA package to analyze the correlation between gene expression and eigengene modules. These networks were visualized using Cytoscape v.3.7.2 software.

## 3 Results

### 3.1 Morphological and cytological comparisons between XHJ and DHJ

**Figure 1:**
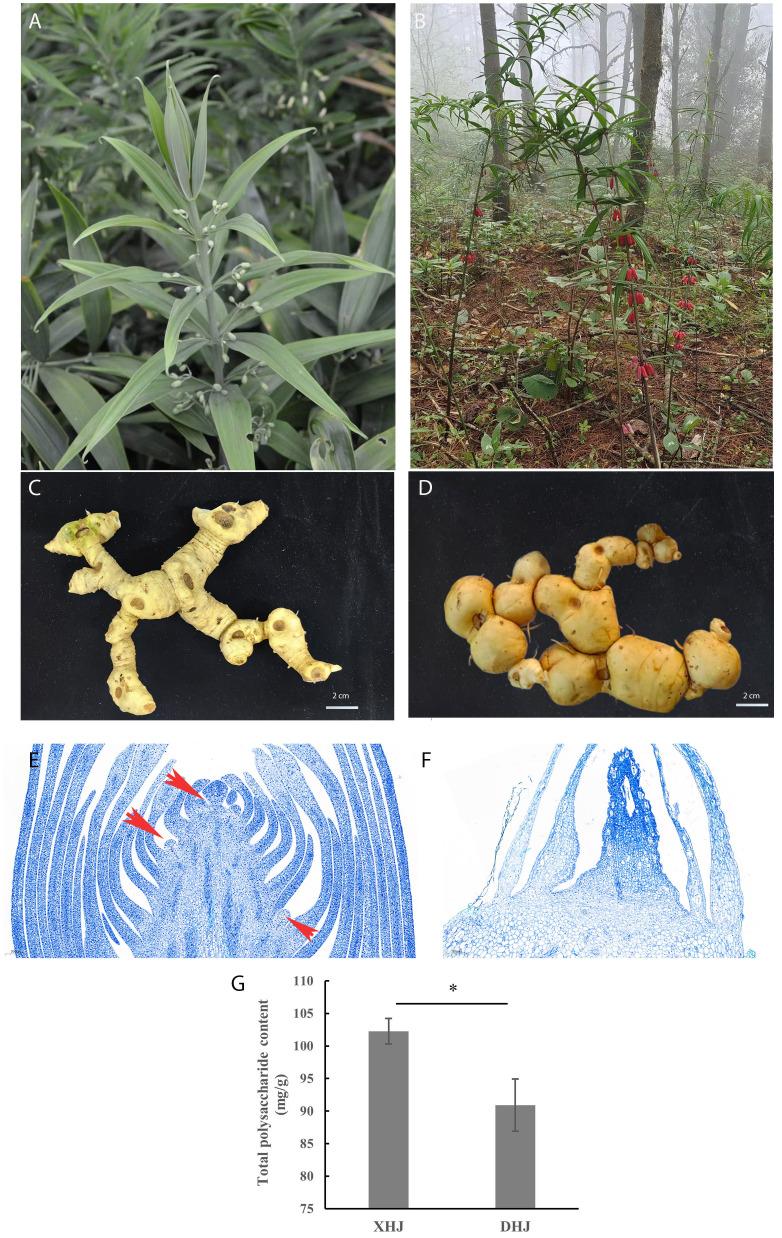
Morphological features of XHJ and DHJ. The overground part of XHJ **(A)** and DHJ **(B)**. The underground rhizome of XHJ **(C)** and DHJ **(D)**; bar = 2 cm. The paraffin section observation of the rhizome buds of XHJ **(E)** and DHJ **(F)** in autumn. Red arrows represent floral primordium. Bar = 200 μm. **(G)** The total polysaccharide content of XHJ and DHJ rhizome. Mg/g on the y-axis was dry weight content (mean ± SD, n≥3, *: p<0.05).

Both XHJ and DHJ belong to the whorled leaf group in *Polygonatum* Mill. However, the flower color of XHJ is greenish-white, while it is red in DHJ (**Figures 1A, B**). The rhizome morphology of XHJ and DHJ was also different (**Figures 1C, D**) as the rhizome node of DHJ was more rounded. The paraffin sections observation showed that the rhizome bud of XHJ in autumn was completely differentiated, which was easy to see in the floral and leaf primordium (**Figure 1E**), but the rhizome bud of DHJ in autumn was undifferentiated and stayed dormant (**Figure 1F**). The total polysaccharide content of XHJ and DHJ rhizome was 102.25 mg/g and 90.91mg/g, respectively (**Figure 1G**).

### 3.2 Phytohormone analysis of XHJ and DHJ

**Figure 2:**
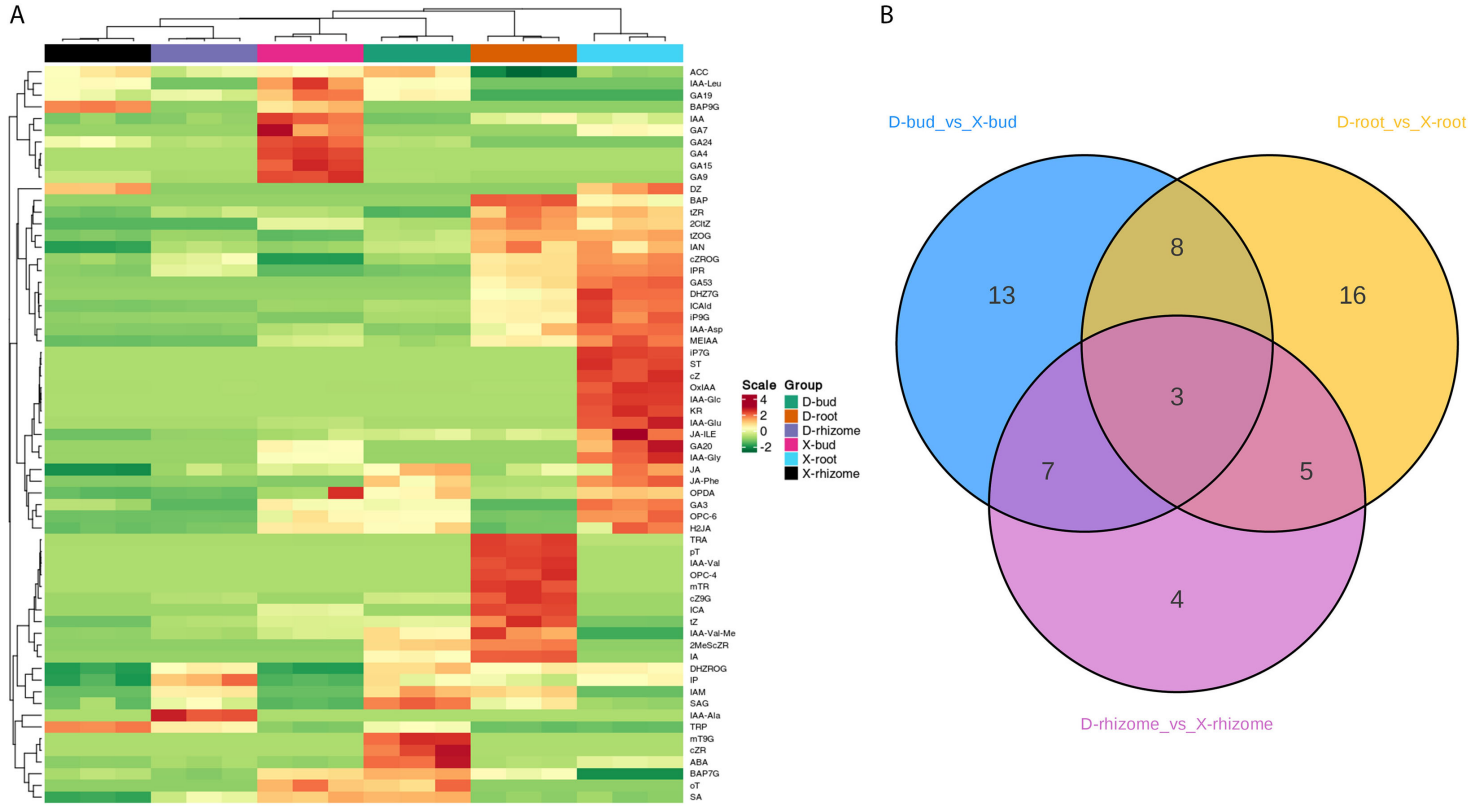
The phytohormones analysis in XHJ and DHJ. **(A)** Heatmap analysis of the relative content of phytohormones in different samples. **(B)** A Venn diagram showing the DAPs in three tissues.3.3 Comparative transcriptomic analysis between XHJ and DHJ.

To fully clarify the different rhizome bud germination mechanisms of XHJ and DHJ in autumn, a targeted phytohormone metabolomic analysis was conducted in XHJ rhizome buds, XHJ rhizomes, XHJ roots, DHJ rhizome buds, DHJ rhizomes, and DHJ roots. Finally, 63 phytohormones were identified in these tissues (**Figure 2A**; **Supplementary Table S2**). The heatmap showed that the X-rhizomes and D-rhizomes were located in the same cluster, the X-buds and D-buds were on the adjacent branch, and the X-roots and D-roots were on another cluster, which indicated that the phytohormones in the same tissues were relatively similar. However, the content of GA19, GA7, GA24, GA4, GA15, and GA9 was significantly higher in the X-buds, and the content of mT9G, cZR, and ABA was significantly higher in the D-buds. The content of IP, IAA-Ala, SA, and SAG was higher in the D-rhizomes. Moreover, the content of iP7G, Strigol, cZ, OxIAA, IAA-Glu, GA20, GA3, JA, JA-ILE, and JA-Phe was particularly higher in the X-roots (**Figure 2A**; **Supplementary Table S2**). Compared with the D-buds, 14 upregulated and 17 downregulated DAPs were identified in the X-buds. Compared with the D-rhizomes, 6 upregulated and 13 downregulated DAPs were identified in the X-rhizomes. Compared with the D-roots, 18 upregulated and 14 downregulated DAPs were identified in the X-roots (**Figure 2B**; **Supplementary Table S3**).

**Figure 3:**
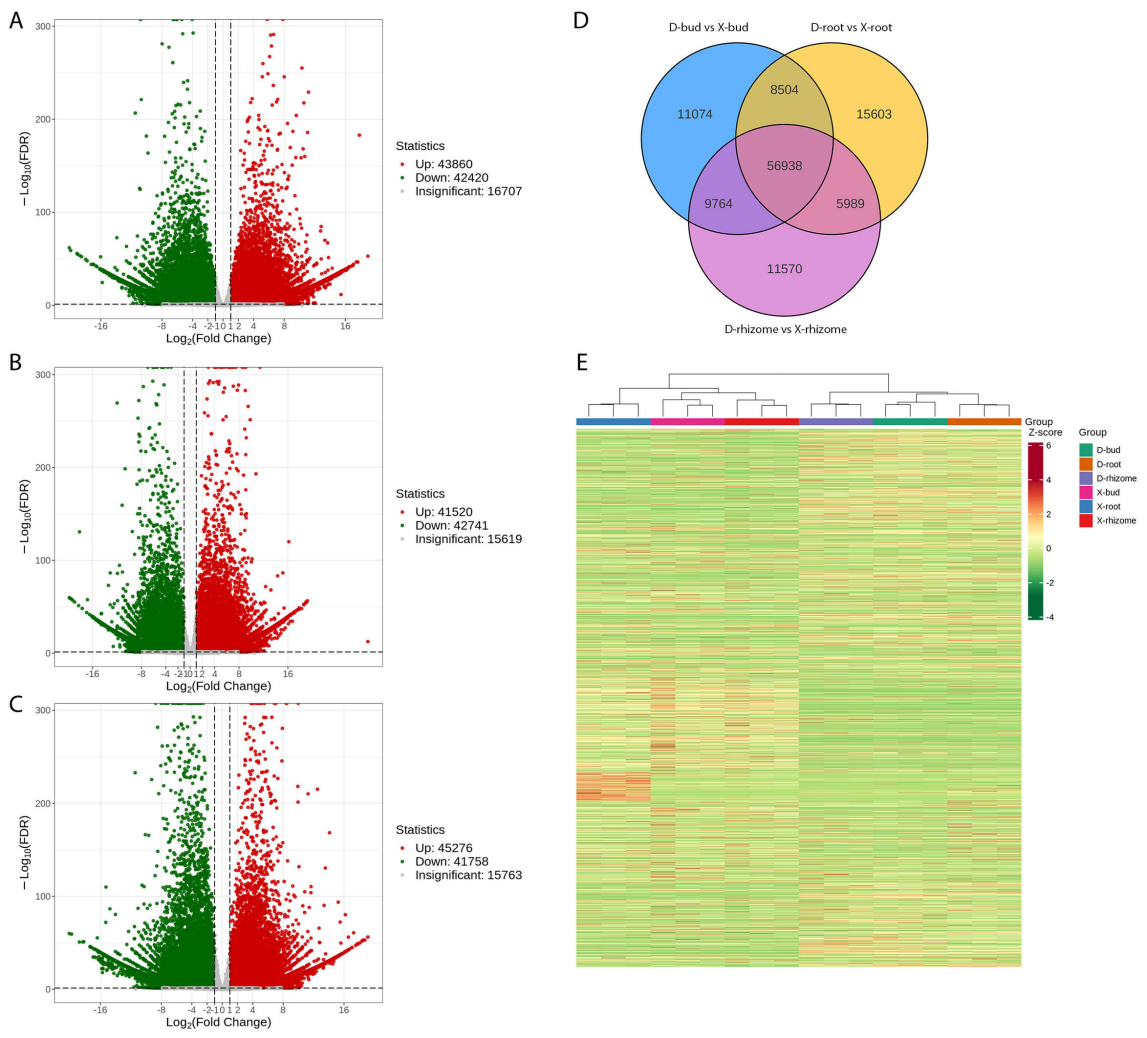
Analysis of DEGs between buds, rhizomes, and roots of XHJ and DHJ. Volcano plot of the DEGs in D-buds vs X-buds **(A)**, D-rhizomes vs X-rhizomes **(B)**, and D-roots vs X-roots **(C)**. **(D)** Venn diagram of DEGs. **(E)** Expression of DEGs in different tissues.

To reveal the different shooting mechanisms between XHJ and DHJ in autumn, RNA-seq of buds, rhizomes, and roots of them were profiled, which generated a total of 818.6 million raw reads (**Supplementary Table S4**). The final assemblies were well annotated and made available. The transcript N50 value was 1159 bp, the unigene N50 value was 1364 bp, and the total BUSCO score was 98.1% (**Supplementary Table S4**). A good repetition among all biological replicates in each group was obtained (**Supplementary Figure S1**). In total, 298,251 unigenes were annotated using the KEGG, NR, Swiss-Prot, GO, COG/KOG, TrEMBL, and Pfam databases (**Supplementary Table S5**). The DEGs were analyzed and a total of 43,860, 41,520, and 45,276 upregulated DEGs, and 42,420, 42,741, and 41,758 downregulated DEGs (P < 0.05) were identified in D-buds vs X-buds, D-rhizomes vs X-rhizomes, and D-roots vs X-roots, respectively (**Figures 3A–C**). There were 11,074, 11,570, and 15,603 DEGs specifically differentially expressed in the buds, rhizomes, and roots, respectively (**Figure 3D**). Hierarchical cluster analysis (HCA) of all the DEGs was conducted to reveal the global gene expression pattern in different groups (**Figure 3E**). As a result, all the XHJ samples were significantly separated from the DHJ samples.

According to the KEGG pathway analysis, in D-buds vs X-buds, ‘Plant hormone signal transduction’ (ko04075) and ‘Photosynthesis - antenna proteins’ (ko00196) were significantly enriched (**Supplementary Table S6**). In D-rhizomes vs X-rhizomes, ‘Ribosome biogenesis in eukaryotes’ (ko03008) and ‘Spliceosome’ (ko03040) were significantly enriched (**Supplementary Table S6**). In D-roots vs X-roots, ‘Proteasome’ (ko03050) and ‘Protein processing in endoplasmic reticulum’ (ko04141) were significantly enriched (**Supplementary Table S6**).

Furthermore, 11 DEGs related to plant hormone metabolism were selected for qRT-PCR validation (**Supplementary Figure S2**), including *Cluster-21187.19* (*YUCCA8-like gene*), *Cluster-65218.0* (*GA20 oxidase 1-D-like*), *Cluster-56959.5* (*GA3-beta-dioxygenase 1-like*), and so on. The expression patterns of the most selected DEGs were consistent with the RNA-seq data, which suggested the RNA-seq data in the present study were reliable.

### 3.4 Correlation analysis between DAPs and DEGs

**Figure 4:**
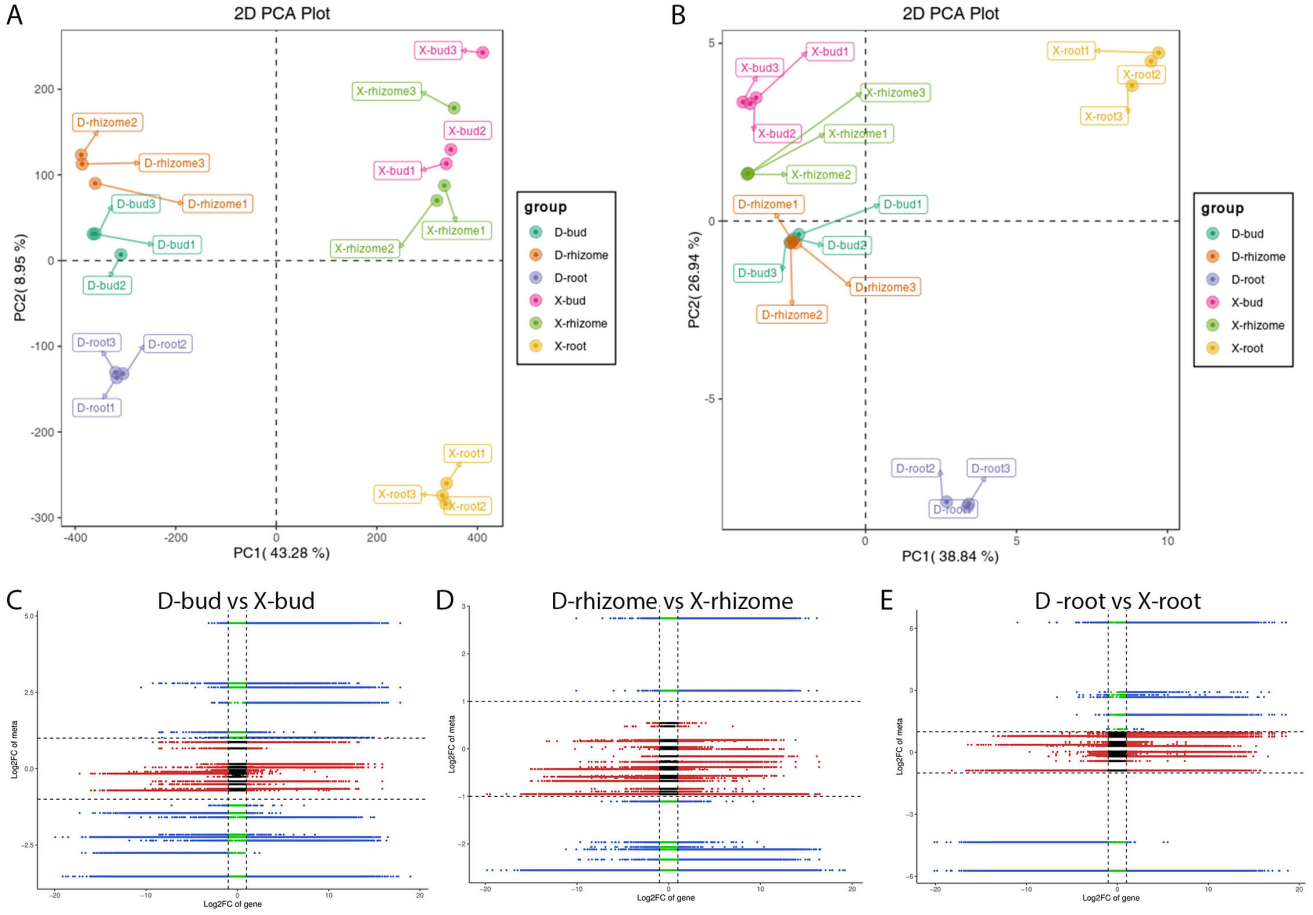
Correlation analysis between DAPs and DEGs. **(A)** PCA of genes in different groups. **(B)** PCA of metabolites in different groups. Nine-quadrant maps of DAPs and DEGs in D-buds vs X-buds **(C)**, D-rhizomes vs X-rhizomes **(D)**, and D-roots vs X-roots **(E)**.

In transcriptome, PCA results showed that PC1 and PC2 explained 43.28% and 8.95% of the total variance, respectively (**Figure 4A**). X-roots and D-roots could be distinguished from X-rhizomes, D-rhizomes, X-buds, and D-buds by PC1 and PC2. X-rhizomes were difficult to distinguish from X-buds by PC1. D-rhizomes were also difficult to distinguish from D-buds by PC1 and PC2 (**Figure 4A**). In the metabolome, the PCA results showed that PC1 and PC2 explained 38.84% and 26.94% of the total variance, respectively (**Figure 4B**). X-roots and D-roots could also be distinguished from other samples by PC1 and PC2. X-rhizomes were difficult to distinguish from X-buds by PC1. D-rhizomes were also difficult to distinguish from D-buds by PC1 and PC2. These results were consistent with the PCA results in the transcriptome (**Figure 4B**). However, the distributions of the X-rhizome, D-rhizome, X-bud, and D-bud groups were more concentrated than those in transcriptome.

**Figure 5:**
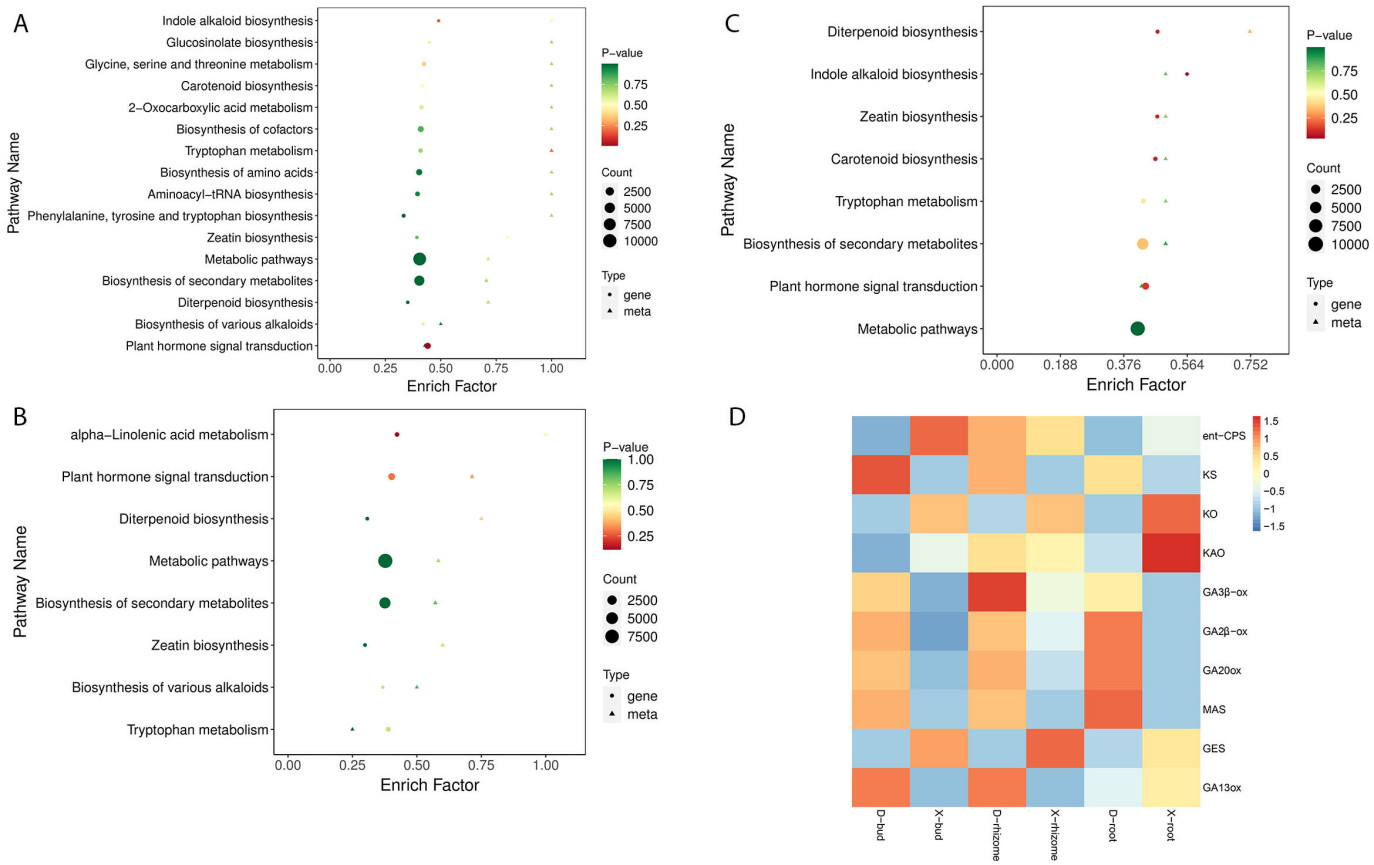
The common KEGG pathways enriched by DAPs and DEGs in D-buds vs X-buds **(A)**, D-rhizomes vs X-rhizomes **(B)**, and D-roots vs X-roots **(C)**. **(D)** The relative expression levels of unigenes in diterpenoid biosynthesis (ko00904). The red color corresponds to higher expression levels. The blue color represents lower expression levels.

To illustrate the relationships between DAPs and DEGs, a nine-quadrant maps were drawn (**Figures 4C–E**). The results showed that most DAPs (36 in D-buds vs X-buds, 35 in D-rhizomes vs X-rhizomes, and 32 in D-roots vs X-roots) and DEGs (18,888 in D-buds vs X-buds, 31,850 in D-rhizomes vs X-rhizomes, and 37,166 in D-roots vs X-roots) were in quadrants 2, 4, 6, and 8, indicating no correlation between these metabolites and genes. DAPs and DEGs in quadrants 3 and 7 were positively correlated (**Figures 4C–E**; **Supplementary Table S7**). In D-buds vs X-buds, 6 DAPs (including tZR, GA9, GA24, IAA, IAA-Leu, and IAA-Asp) and 15,339 DEGs were up-regulated in quadrant 3, and 13 DAPs (including ABA, 3-Indole acetamide, salicylic acid 2-O-β-glucoside, and so on) and 49,478 DEGs were downregulated in quadrant 7 (**Figure 4C**; **Supplementary Table S7**). In D-rhizomes vs X-rhizomes, 4 DAPs (including GA3, GA9, GA24, and IAA-Leu) and 7,997 DEGs were upregulated in quadrant 3, and 12 DAPs (including SA, JA, JA-ILE, trans-zeatin (tZ), and so on) and 30,857 DEGs were downregulated in quadrant 7 (**Figure 4D**; **Supplementary Table S7**). In D-roots vs X-roots, 7 DAPs (including GA3, OxIAA, JA-Phe and so on) and 9,607 DEGs were upregulated in quadrant 3, and 6 DAPs (including tZ, cZ9G, TRA, and so on) and 44,327 DEGs were downregulated in quadrant 7 (**Figure 4E**; **Supplementary Table S7**). The common KEGG pathways enriched by the DEGs and DAPs are shown in **Figures 5A–C** (**Supplementary Table S8**). Interestingly, ‘Diterpenoid biosynthesis’ (ko00904) and ‘Plant hormone signal transduction’ (ko04075) were collectively enriched in all tissues. Among these DAPs, GAs were significantly different between XHJ and DHJ. As is well known, GAs belong to ‘Diterpenoid’, so the DEGs involved in diterpenoid metabolism were further focused on. In ‘Diterpenoid biosynthesis’ (ko00904), the relative expression levels of the related DEGs are shown in **Figure 5D**, including the *ent-copalyl diphosphate synthase gene* (*ent-CPS*), *ent-kaurene synthas*e gene (*KS*), *ent-kaurene oxidase gene* (*KO*), *ent-kaurenoic acid oxidase gene* (*KAO*), *GA 3β-dioxygenase gene* (*GA3β-*ox), *GA 2β-dioxygenase gene* (GA*2β-*ox), *GA20 oxidase gene* (*GA20ox*), *momilactone-A synthase gene* (*MAS*), *geranyllinalool synthase gene* (*GES*), and *GA13 oxidase gene* (*GA13ox*). Among these, *ent-CPS*, *KO*, *KAO*, and *GES* showed relatively higher expression in XHJ, while *KS*, *MAS*, *GA3β-ox*, *GA2β-ox*, *GA20ox*, and *GA13ox* were upregulated in DHJ (**Figure 5D**). In addition, the relative expression of unigenes related to ETH and SL metabolites were upregulated in XHJ, such as *ACC oxidase gene*, *ACC synthase* gene, *DWARF27 (D27) isomerase gene*, *Carotenoid cleavage dioxygenase 7* (*CCD7*), and *CCD8* (**Supplementary Table S9**).

### 3.5 Co-expression network analysis

**Figure 6:**
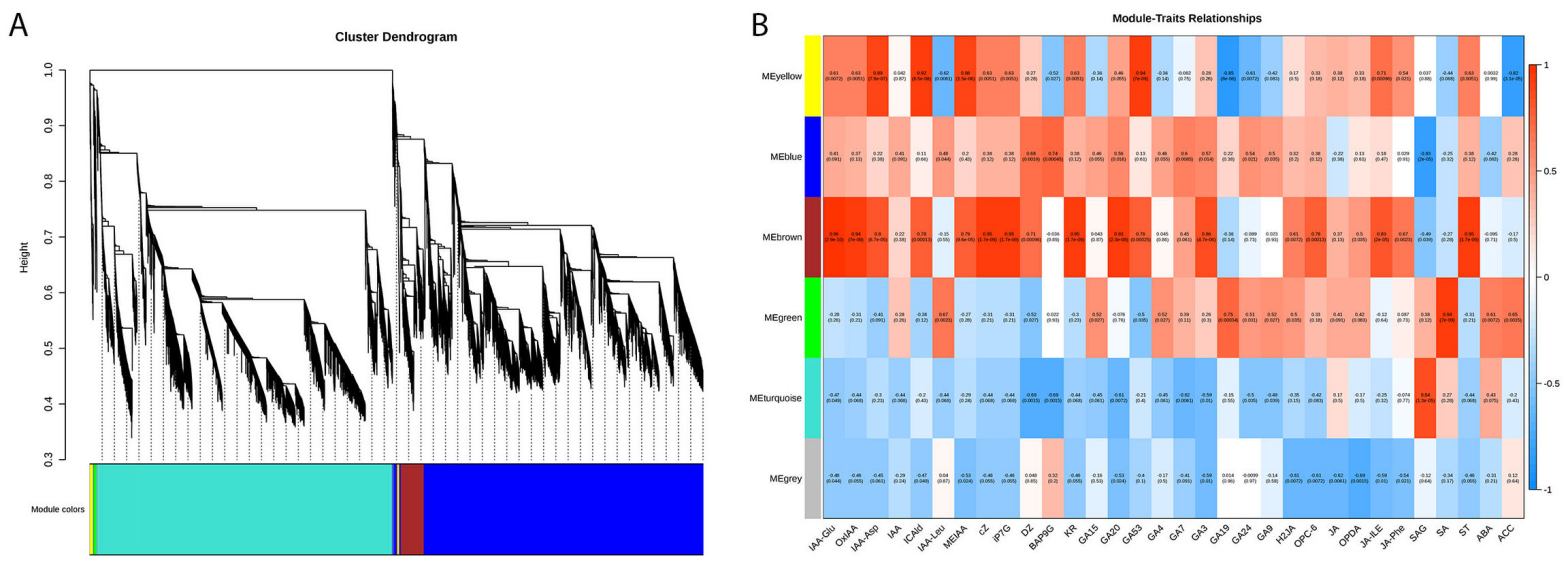
Metabolic and transcriptomic correlation analysis in XHJ and DHJ. **(A)** Dendrogram showing co-expression modules identified by WGCNA. The major two branches constitute six modules labeled with different colors. **(B)** Heatmap of the module-metabolite correlations. Each row corresponds to a module indicated by different colors. Each column corresponds to a metabolite. The blue color represents a negative correlation. The red color represents a positive correlation.

To gain further insight into the gene regulatory network and identify potential key regulatory factors related to auxin, CTK, GA, and JA metabolism in XHJ and DHJ, a WGCNA was conducted. A total of six co-expression modules were identified according to similar expression patterns (**Figure 6A**; **Supplementary Table S10**). The heatmap of module-trait correlations showed the accumulation of transcripts in the brown module was highly correlated with most of the phytohormones, such as IAA-Glu, GA20, and GA3 (**Figure 6B**).

**Figure 7:**
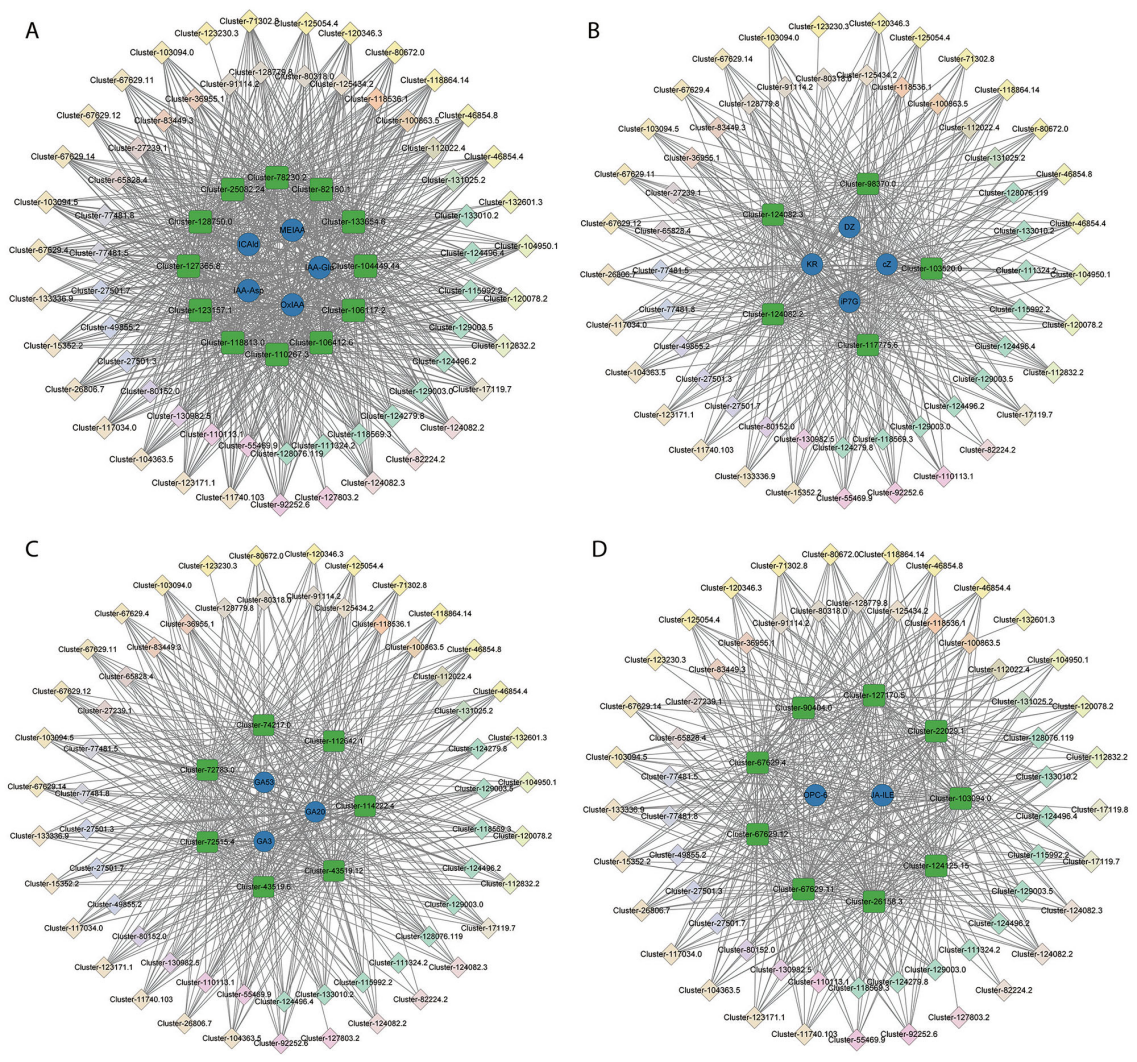
The regulatory network of auxin **(A)**, CTK **(B)**, GA **(C)**, and JA **(D)** in the brown module. The blue circles represent phytohormones. Green squares represent structural genes related to the relative phytohormones. The diamond represents TFs identified in the same module whose transcripts are correlated with the expression of structural genes, and different colors represent different TF families.Additionally, seven structural genes related to GA metabolic processes in the brown module were identified, including two GA receptor *GID1* (*Cluster-72515.4* and *Cluster-74217.0*), two *KO* (*Cluster-43519.12* and *Cluster-43519.6*), *GA2β-ox* (*Cluster-72783.0*), *KAO* (*Cluster-112642.1*), and *MAS* (*Cluster-114222.4*), which were related to the GA biosynthetic process (GO:0009686) (**Supplementary Table S11**). These genes had a high correlation with the accumulation of GA20, GA53, and GA3. Furthermore, 62 (the top three gene families were *AP2/ERFs*, *WRKY*, and *NAC*) TFs were identified to be highly correlated with the seven GA metabolism-related structural genes in the brown module and we constructed a correlation network (**Figure 7C**; **Supplementary Table S12**).

To generate the potential regulatory network associated with phytohormone metabolism, the structural genes in the brown module were further analyzed (absolute value of Pearson’s correlation coefficient > 0.9, p-value < 0.01) (**Figure 6A**; **Supplementary Table S10**). We identified 12 structural genes related to the auxin metabolic process in the brown module, including *Cluster-133654.6* (auxin efflux carrier family protein: *PIN-LIKES 3-like*), *Cluster-78230.2* (SAUR family protein: *auxin-induced protein 6B-like*), *Cluster-106117.2* (GO:2000012, regulation of auxin polar transport), and so on (**Supplementary Table S11**), which had a high correlation with the accumulation of IAA-Asp, IAA-Glu, ICAld, MEIAA, and OxIAA. By correlating the accumulation patterns of transcripts and the potential binding affinity of the structural genes associated with auxin metabolism, we identified 62 TFs (the top three gene families were *AP2/ERFs*, *WRKY*, and *NAC*), whose expression was highly correlated with the 12 auxin-related structural genes in the brown module and constructed a correlation network (**Figure 7A**; **Supplementary Table S12**).

In addition, five CTK metabolism-related structural genes were identified, including two CTK-activated signaling pathway genes (GO:0009736: *Cluster-103520.0* and *Cluster-117775.6*), two gene responses to CTK (GO:0009735: *Cluster-124082.2* and *Cluster-124082.3*), and one *CTK dehydrogenase gene* (*CKX*, *Cluster-98370.0*), which had a high correlation with the accumulation of cZ, iP7G, DZ, and KR (**Supplementary Table S11**). Additionally, 58 TFs (the top three gene families were *AP2/ERFs*, *WRKY*, and *NAC*) were identified with a high correlation with the five CTK metabolism-related structural genes in the brown module and we constructed a correlation network (**Figure 7B**; **Supplementary Table S12**).

Moreover, nine structural genes related to the JA metabolic process in the brown module were identified, including four JA ZIM domain-containing proteins (*Cluster-103094.0*, *Cluster-67629.11*, *Cluster-67629.12* and *Cluster-67629.4*), one JA-induced oxygenase 2 (*Cluster-124125.15*), one JA methylesterase (*Cluster-127170.5*), two JA-induced proteins (*Cluster-22029.1* and *Cluster-26158.3*), and one JA O-methyltransferase (*Cluster-90404.0*), which had a high correlation with the accumulation of OPC-6, JA-ILE, and JA-Phe (**Supplementary Table S11**). Furthermore, 59 TFs (such as *AP2/ERFs*, *WRKY*, and *NAC*) were identified to be highly correlated with the nine JA metabolism-related structural genes in the brown module and we constructed a correlation network (**Figure 7D**; **Supplementary Table S12**).

## 4 Discussion

Huangjing does not contain starch but is rich in nutrients such as fructans with easy degradation and energy, which is important for health and serving life. Additionally, Huangjing is suitable for understory planting or intercropping with maize and does not take up farmland or forest land. It thus has enormous production capacity and is vital for ensuring food security (Si et al., 2024). However, the Huangjing industry also has faced multiple challenges, such as weak basic research.

XHJ is a variant of DHJ. The total polysaccharide content of XHJ was significantly higher than in DHJ (**Figure 1G**), which indicated that XHJ maintained a high content of active ingredients while maintaining a high yield. Moreover, the morphological features of XHJ and DHJ were a little different (**Figures 1A–D**), especially in the bud germination habits. According to our cellular observation (**Figures 1E, F**), the status of X-buds and D-buds in autumn were significantly different. We found no differentiation signs of D-buds in autumn. However, it was easy to see the floral and leaf primordium in the X-buds, which germinate and generate leaves and flowers. It was interesting that the X-buds and D-buds in the present study were newly born in the current year, while the X-buds seemed to have no need for dormancy or had a short dormancy in summer. This phenomenon is similar to the shooting habit in *C. pingbianense*, a kind of woody bamboo that appears to have a natural four-season shooting (other bamboos only germinate in spring) (Li L. et al., 2022). Hence, our next step will be to maintain constant cytological observation of bud formation and development all year round to further uncover the rhizome bud germination features of XHJ.

In the phytohormone analysis, 63 phytohormones were identified in the buds, rhizomes, and roots of XHJ and DHJ (**Figure 2A**; **Supplementary Table S2**). According to the heatmap, the same tissues were clustered closely, but the content of a few phytohormones was significantly different. In strawberries and DHJ, the dormant buds contained higher IAA content than the non-dormant buds (Wang et al., 2019; Qiu et al., 2019). However, we found the auxin concentration was relatively higher in the X-buds, especially IAA and IAA-Leu, which was consistent with the results in *C. pingbianense*. The all-year high IAA content in rhizome buds may contribute to its natural four-season shooting (Mao et al., 2024). Moreover, *PIN-LIKES 3-like* (*Cluster-133654.6*), *SAUR* (*Cluster-78230.2*), and *glutathione S-transferase* (*Cluster-110267.3*), which are important for auxin transport and response (Li et al., 2017; Adamowski and Friml, 2015; Smith et al., 2003), were obviously upregulated in XHJ (**Figure 7A**; **Supplementary Table S11**), which was consistent with the phytohormone analysis (**Figure 2A**). These results imply that the germination of X-buds in autumn might be associated with the strong metabolism of auxin, but this requires further study.

In a previous study, dormant buds contained 1.6-fold lower trans-zeatin than non-dormant buds in strawberries (Qiu et al., 2019). In apple trees, CTK was often applied to generate more shoot branches in apple seedlings, and the axillary bud activation and outgrowth were dependent on local CTK (Li et al., 2018). In *Oryza longistaminata*, the CTK biosynthesis genes were upregulated with accompanying CTK accumulation in the early stages of the response to nitrogen application, which was capable of promoting rhizome bud outgrowth to produce the secondary rhizome (Shibasaki et al., 2021). In addition, the endo-dormancy to non-dormancy transition of the DHJ rhizome buds was also related to an increased tZ level (Wang et al., 2019). These results suggest that CTK benefits axillary bud and rhizome bud development and releases dormancy in these species. However, we found the tZ content in the D-buds was significantly higher than the X-buds (**Figure 2**; **Supplementary Table S2**), and the *CTK dehydrogenase gene* (*CKX*: *Cluster-98370.0*) was relatively upregulated in XHJ (**Figure 7A**; **Supplementary Table S11**). CKX catalyzes the irreversible breakdown of active CTK, and the CTK levels were significantly increased in the *osckx11* mutant when compared with the wild-type in rice (Zhang W. et al., 2021; Chen et al., 2020). In our study, tZ showed the opposite change compared with previous studies, which might imply that the regulation modules of CTK in XHJ are different from other species, and the underlying mechanism for this phenomenon deserves further study.

In phytohormone analysis, the content of GA was relatively higher in the roots, buds, and rhizomes of XHJ when compared with those of DHJ, while the ABA content in D-buds was the highest (**Figure 2**; **Supplementary Table S2**). These results indicate that the ABA/GA ratio in the X-buds was lower than that in the D-buds, which is consistent with the reduced ABA/GA3 ratio in the transition of rhizome buds from endo-dormancy to non-dormancy in DHJ (Wang et al., 2019) and many other species, such as *C. pingbianense*, *P. pyrifolia*, and *P. lactiflora* (Yang et al., 2023; Li L. et al., 2022; Zhang R. L. et al., 2021). According to our transcriptome results, the ‘diterpenoid biosynthesis’ (ko00904) pathway was enriched in all tissues, and *KO* and *KAO* were upregulated in XHJ (**Figure 5**). In *Arabidopsis*, KO catalyzes three steps of GA biosynthesis (Helliwell et al., 1999). In addition, KAO catalyzes the conversion of ent-kaurenoic acid to GA12, which is the precursor of all GAs, thereby playing a crucial role in determining GA concentration in plants (Regnault et al., 2014). However, *KS, MAS, GA3β-ox, GA2β-ox, GA20ox*, and *GA13ox* were also important for GA metabolism and were upregulated in DHJ in the present study, while the GA content in DHJ was relatively lower. This might be because of the post-translational modification and these genes’ expression results were inconsistent with the GA content. Interestingly, the ABA/GA ratio in X-buds was significantly lower than in the other samples (**Supplementary Table S2**), which suggests that a low ABA/GA ratio might contribute to the germination of X-buds in autumn.

Furthermore, the relative content of JAs in X-roots and D-buds was obviously higher than those in other samples (**Figure 2**; **Supplementary Table S2**). In plants, JAs are important signaling compounds implicated in plant development and stress defense (Ghorbel et al., 2021). Moreover, JAs also play an inhibitory role in *Arabidopsis* bud formation, which suppresses *FT* expression and delays flowering through the JAZ signaling pathway (Wang et al., 2017; Zhai et al., 2015). Here, we found no differentiation signs in the floral primordium in D-buds in autumn (**Figure 1D**), which was consistent with its relatively higher JA content. Hence, whether the low JA content in X-buds contributes to its germination in autumn also requires further research.

Additionally, the content of SA and ETH in D-buds was also relatively higher than that in X-buds (**Figure 2**; **Supplementary Table S2**). A previous report suggested SA inhibits the production of α-amylase by decreasing GA-mediated α-amylase expression during rice germination (Xie et al., 2007). Recently, a significant enhancement of SA might participate in the rhubarb bud dormancy development (Wojtania et al., 2022). In *Vitis vinifera*, ETH signaling appeared critical for bud dormancy release (Shi et al., 2020; Ophir et al., 2009). The levels of ETH and ACC oxidase transcripts sharply decrease during the natural dormancy release of grapevine buds, whereas ACC accumulates (Shi et al., 2018). The present study showed most ACC oxidase and ACC synthase transcripts (including 28 unigenes) were upregulated in XHJ (**Supplementary Table S9**), while the level of ACC in D-buds was the highest, which might be because the post-translational modification affected the function of ACC oxidase and ACC synthase.

In our study, Strigol (a type of SL) was only detected in X-roots (**Figure 2A**; **Supplementary Table S2**). SL is a type of root-derived chemical signal that regulates diverse developmental processes and environmental responses in plants (Mashiguchi et al., 2021). SL inhibited the bud growth in Arabidopsis and mediated axillary bud dormancy in rice (Lantzouni et al., 2017; Luo et al., 2019). Furthermore, most *D27 isomerase genes* and *CCD7* and *CCD8* in the SL biosynthetic pathway were significantly upregulated in X-roots (**Supplementary Table S9**). Whether the high level of SL in X-roots contributed to X-bud germination in autumn also deserves further research.

In the WGCNA, TFs related to the auxin, CTK, GA, and JA metabolites were identified, such as *AP2/ERF-ERF*, *WRKY*, and *NAC* (**Figure 7**; **Supplementary Table S12**). *AP2/ERFs* are a big gene family that is mostly found in plants and are usually a crucial regulator in flowering (Maes et al., 2001). In *Chrysanthemum morifolium*, *ERF*s were related to the regulation of photoperiodic flowering, and the overexpression of *CmERF110* led to earlier flowering (Huang et al., 2022). In the present study, the flower differentiation of DHJ buds in autumn was inhibited (**Figure 1F**), which might be involved in their relatively low expression in DHJ when compared with XHJ (**Supplementary Table S12**). Additionally, the regulation of *WRKY* in bud germination has been previously reported in different plant species, including peach, grape, and Arabidopsis (Wang et al., 2022; Chen et al., 2016; Guo et al., 2015). *NAC* is one of the largest plant-specific gene families and plays significant roles in the regulation of plant growth and development, disease resistance, and stress response (Yu et al., 2023). In the present study, the selected *AP2/ERF*, *WRKY*, and *NAC* TFs in all the correlation networks were significantly upregulated in XHJ (**Supplementary Table S12**), and are candidate genes that deserve further study to uncover the potential mechanism of X-bud autumn shooting.

## 5 Conclusion

In the present study, cellular observation, comparative targeted metabolome of phytohormones, and transcriptome analysis were conducted to uncover the autumn shooting mechanisms of XHJ by comparing it with DHJ. ‘Diterpenoid biosynthesis’ (ko00904) and ‘Plant hormone signal transduction’ (ko04075) were commonly enriched by the DAPs and DEGs in all tissues. In this study, a low ABA/GA ratio and the metabolism of auxin and CTK might contribute to the XHJ rhizome buds’ differentiation and germination in autumn, which might be related to the regulation of TFs such as *AP2/ERFs*, *WRKY*, and *NAC*. Our study comprehensively illustrates the mechanism of XHJ rhizome bud germination in autumn and improves our understanding of the diversity of shooting mechanisms in *Polygonatum* to lay the foundation for the further development of the Huangjing industry.

## Data Availability

The datasets presented in this study can be found in online repositories. The names of the repository/repositories and accession number(s) can be found in the article/**Supplementary Material**.
