# Supplementary figures and images for: An integrated targeted metabolome of phytohormones and transcriptomics analysis provides insight into the new generation of crops: Polygonatum kingianum var. grandifolium and Polygonatum kingianum
Source: Front Plant Sci. 2024 Sep 24;15:1464731. doi: 10.3389/fpls.2024.1464731 (PMC11458434; doi:10.3389/fpls.2024.1464731)

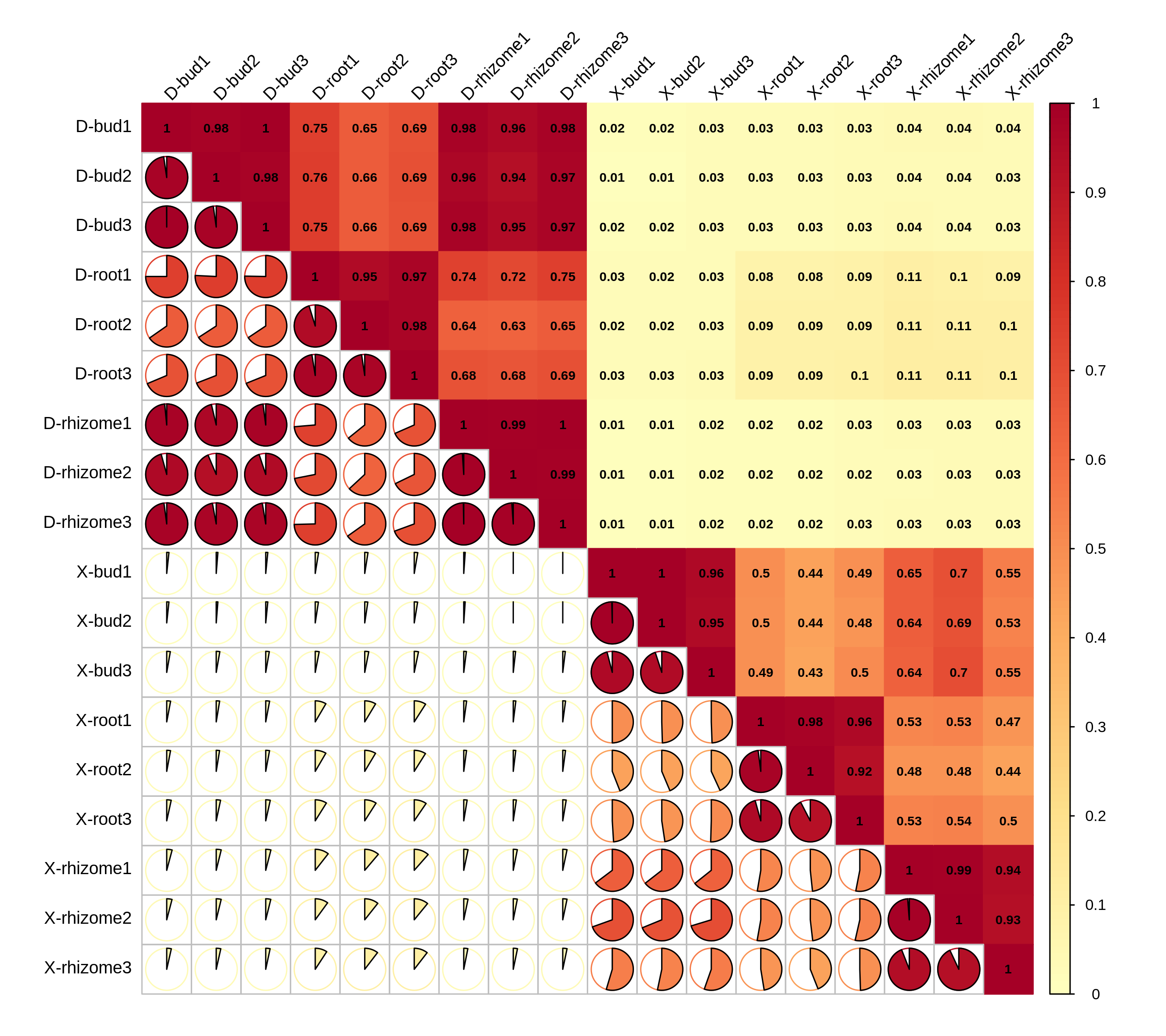

Supplement: Supplementary file 1 [file Image1.png]

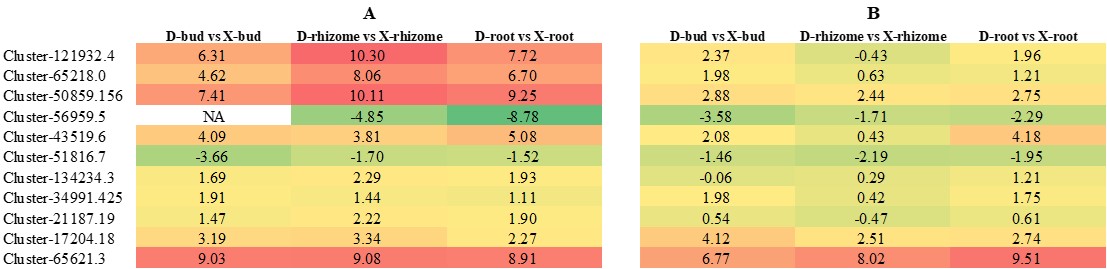

Supplement: Supplementary file 2 [file Image2.jpeg]
